# The progressive nature of Wallerian degeneration in wild-type and slow Wallerian degeneration (Wld^S^) nerves

**DOI:** 10.1186/1471-2202-6-6

**Published:** 2005-02-01

**Authors:** Bogdan Beirowski, Robert Adalbert, Diana Wagner, Daniela S Grumme, Klaus Addicks, Richard R Ribchester, Michael P Coleman

**Affiliations:** 1 Center for Molecular Medicine Cologne (CMMC) and Institute for Genetics, University of Cologne, Zuelpicher Strasse 47, D-50647 Cologne, Germany; 2 Department of Anatomy I, University of Cologne, Joseph-Stelzmann Strasse 9, D-50931 Cologne, Germany; 3 Division of Neuroscience, University of Edinburgh, 1 George Square, Edinburgh, EH8 9JZ, UK; 4 Babraham Institute, Babraham, Cambridge CB2 4 AT, UK

## Abstract

**Background:**

The progressive nature of Wallerian degeneration has long been controversial. Conflicting reports that distal stumps of injured axons degenerate anterogradely, retrogradely, or simultaneously are based on statistical observations at discontinuous locations within the nerve, without observing any single axon at two distant points. As axon degeneration is asynchronous, there are clear advantages to longitudinal studies of individual degenerating axons. We recently validated the study of Wallerian degeneration using yellow fluorescent protein (YFP) in a small, representative population of axons, which greatly improves longitudinal imaging. Here, we apply this method to study the progressive nature of Wallerian degeneration in both wild-type and slow Wallerian degeneration (Wld^S^) mutant mice.

**Results:**

In wild-type nerves, we directly observed partially fragmented axons (average 5.3%) among a majority of fully intact or degenerated axons 37–42 h after transection and 40–44 h after crush injury. Axons exist in this state only transiently, probably for less than one hour. Surprisingly, axons degenerated anterogradely after transection but retrogradely after a crush, but in both cases a sharp boundary separated intact and fragmented regions of individual axons, indicating that Wallerian degeneration progresses as a wave sequentially affecting adjacent regions of the axon. In contrast, most or all Wld^S ^axons were partially fragmented 15–25 days after nerve lesion, Wld^S ^axons degenerated anterogradely independent of lesion type, and signs of degeneration increased gradually along the nerve instead of abruptly. Furthermore, the first signs of degeneration were short constrictions, not complete breaks.

**Conclusions:**

We conclude that Wallerian degeneration progresses rapidly along individual wild-type axons after a heterogeneous latent phase. The speed of progression and its ability to travel in either direction challenges earlier models in which clearance of trophic or regulatory factors by axonal transport triggers degeneration. Wld^S ^axons, once they finally degenerate, do so by a fundamentally different mechanism, indicated by differences in the rate, direction and abruptness of progression, and by different early morphological signs of degeneration. These observations suggest that Wld^S ^axons undergo a slow anterograde decay as axonal components are gradually depleted, and do not simply follow the degeneration pathway of wild-type axons at a slower rate.

## Background

Wallerian degeneration, the characteristic degeneration sequence of nerve fibres separated from their cell bodies, was described by Waller in 1850 [1,2]. Following various forms of axon injury this rapid degeneration process begins with degradation of axoplasm and axolemma accompanied by development of axonal and myelin debris that is subsequently removed by Schwann cells and invading macrophages. In recent years it became apparent that Wallerian degeneration is initiated by an active process intrinsic to the axon that shares some principles with apoptosis [3-7]. These discoveries were firmly established by studies on the slow Wallerian degeneration (Wld^S^) mutant mouse, in which this active process seems to be turned off. Accordingly, this mutant shows a tenfold delay in Wallerian degeneration and synapse breakdown after experimental nerve injury [8,9]. The delay of Wallerian degeneration is an intrinsic property of the axon suggesting that glial cell and macrophage changes are secondary events [3]. The underlying trait is carried by the autosomal dominant mutation *Wld*^*S *^that arose by spontaneous mutation [6,10]. Genetic analysis has shown that the *Wld*^*S *^mutation on mouse chromosome 4 comprises a stable 85-kb tandem triplication [11,12] encoding the N-terminal 70 amino acids of the multiubiquitination factor Ube4b fused in frame to the nuclear NAD producing enzyme nicotinamide mononucleotide adenylyltransferase 1 (Nmnat 1). Correspondingly, Wld^S ^mice express a novel chimeric protein (Wld^S ^protein) in neuronal nuclei that has full Nmnat 1 activity but seemingly no Ube4b function since the expressed N-terminus lacks ability for multi-ubiquitination within the ubiquitin-proteasome system (UPS) [13,14], the molecular machinery responsible for a major pathway of cellular protein catabolism. Either only one or both parts of the nuclear Wld^S ^protein could be responsible for the phenotype through a nuclear process that has an indirect effect on the axon although recent results foster that the Wld^S ^mechanism is likely to involve a gain of function of NAD synthesis [15]. However, inhibition of a specific step of the ubiquitin proteasome system or another modifying role of the N-terminal domain of the Wld^S ^protein remains a possibility [16,17].

From a clinical point of view not only traumatic disorders such as nerve, spinal cord or head injury result in Wallerian degeneration [18] but it is now broadly accepted that Wallerian degeneration is mechanistically related to axon loss in many neurodegenerative disorders such as amyotrophic lateral sclerosis, Charcot-Marie-Tooth disease, toxic neuropathy, multiple sclerosis, and possibly Alzheimer's Disease and Parkinson's Disease [7,14,19-23]. Protection from neurodegenerative disorders by Wld^S ^is currently under intense investigation. The neuroprotective mutation alleviates diverse PNS axon disorders, including dysmyelination and dying back neuropathy in P0^-/- ^mutants [24], motor neuropathy in *pmn *mutants [25] and axon degeneration in Vincristine and Taxol toxicity [26-28]. More recently Wld^S ^was reported to attenuate pathology in acute CNS lesions caused by stroke [29], Parkinson's disease [30], and in gracile axonal dystrophy (*gad*) mice, a CNS axonal spheroid pathology [31]. A better understanding of the biological mechanism of delayed axon degeneration in neurological diseases would help to develop therapeutic methods to target axon degeneration.

Despite research extending over more than 150 years and its frequent use as tool to detect interneuronal connections in the CNS since the time of Cajal, fundamental issues of Wallerian degeneration remain unresolved and controversial even on a purely morphological level. Among these is the spatiotemporal pattern of Wallerian degeneration along the separated nerve stump. Understanding the exact pattern of spread should provide additional insights into the mechanisms of axon death and may indicate strategies to alter Wallerian degeneration in neurological disease. Shortly after the pioneering investigations of Waller, and in the following decades, there has been much debate as to whether degeneration occurs in an anterograde direction, a retrograde direction or simultaneously along the separated nerve stump axons (reviewed historically in [32-34]).

The controversy over the directionality of Wallerian degeneration has arisen partly because appropriate methods to follow axons over considerable distances did not exist until recently but also because the course of Wallerian degeneration varies with many experimental factors. Thus, spatiotemporal evolution of Wallerian degeneration depends on the laboratory animals used [32,34-36], on the age of the animals [32,37], on the neuroanatomical locus of study (CNS vs. PNS) [35,38,39], on the type of fibre analyzed (e.g. myelinated vs. unmyelinated, thick vs. thin axons) [34,39,40], on the type of lesion (axotomy, crush, ligature, intoxication etc.) [34,41,42], on the length of the remaining distal nerve stump [32,43-45], on the criteria used for identification of fibre degeneration (e.g. myelin breakdown, axon disintegration, decay of electrophysiological activity) [32,33,39], on environmental factors (e.g. temperature) [6,32,34,36] and many more. For example, in more modern experimentation from the last decades, Lubinska [46] demonstrated with the help of the teased fibre technique on myelinated fibres of the rat phrenic nerve that axonal breakdown into myelin ovoids spreads anterogradely along axons separated from their cell bodies at velocities correlated with fibre diameter and internodal length. George and Griffin [47] also found anterograde spread of axonal disintegration along dorsal columns of the rat following L_4_L_5_L_6 _radiculotomy. Contrary to these views, Lunn and colleagues [48] showed by means of silver-stained wholemount preparations from the peripheral nerve stump that degeneration after crushing proceeds in a retrograde direction. They also proposed a retrograde progression after sectioning, freezing or ligaturing, although this was less clear because degeneration was more complete at the time-point sampled. Electrophysiological approaches suggested that the spread of failure of conduction in degenerating mammalian nerves runs from proximal to distal after nerve transection [49]. In cell culture studies using dorsal root ganglions (DRG) explants membrane beading, blebbing, fragmentation and Annexin V staining progressed along interrupted neurites in an anterograde direction with a rate comparable to that of slow axonal transport [50]. Taking secondary changes after axon disintegration into consideration, Bendszus and colleagues [51] tracked an anterograde spatiotemporal course of macrophage infiltration after acute peripheral nerve injury in rats. While most of the above mentioned investigators concluded a progressive nature of Wallerian degeneration from the appearance of degeneration gradients along injured nerves other authors did not observe any evidence for an anterograde or retrograde pattern of axonal degeneration [52,53].

In view of the contradictions and anomalies in the previous literature, we have reassessed the directionality of Wallerian degeneration using a recently introduced technique to visualize individual fluorescent axons over cm-long distances during degeneration [54]. This was made possible by using nerves from transgenic mice expressing Yellow Fluorescent Protein (YFP) in representative subsets of axons, which presents a simplified image of peripheral nerve [55]. No method existed until recently to follow up individual axons undergoing Wallerian degeneration over a considerable length. Here we compared the progression of Wallerian degeneration along single axons traced over lengths of approximately 2.5 cm. Specifically, we have tested a key prediction of all progressive models: that it should be possible to image axons degenerated at one end but not at the other. We detected such axons and showed that Wallerian degeneration in wild-type peripheral nerve is a rapid, asynchronous, progressive and wave-like process that can change its orientation depending on the lesion type.

To our knowledge there have been no reports about the spatiotemporal pattern of the much-delayed axon degeneration in peripheral Wld^S ^nerves that could yield important clues for understanding classic Wallerian degeneration. Therefore, we also report a detailed characterisation of injury-induced axon degeneration in slow Wallerian degeneration mutant mice in order to determine whether axons degenerate with a similar spatial evolution to that in wild-type mice, but in "slow motion", or whether the process is fundamentally distinct. We report a series of differences between axon degeneration in wild-type and Wld^S ^mice, suggesting that irreversible injury in axons where Wallerian degeneration is blocked eventually leads to a different pathway of degeneration.

## Results

### YFP labelled wild-type axons fragment abruptly and asynchronously after a latent phase of approximately 36–44 h

**Figure 1:**
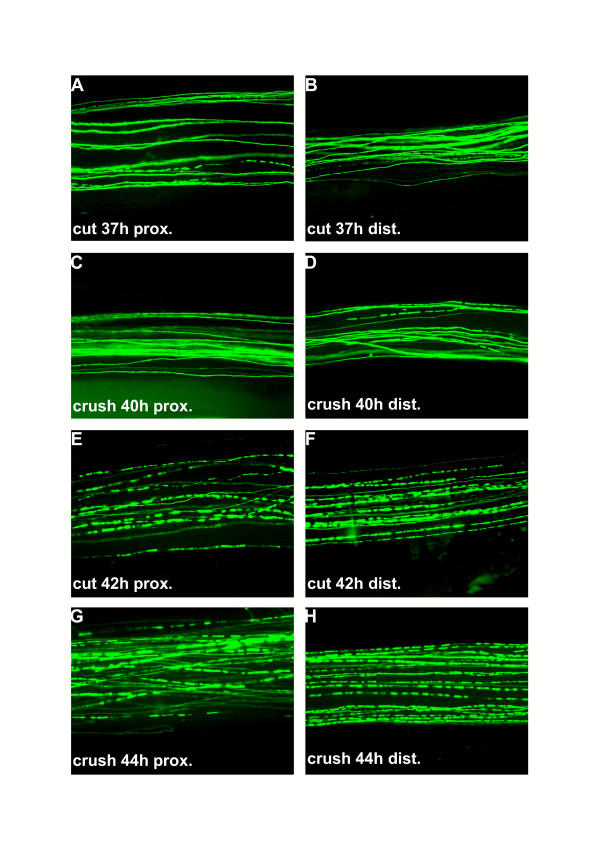
**After a latency period Wallerian degeneration following cut and crush injury starts abruptly in single axons and involves total fragmentation of axons within few hours A-D**: Conventional fluorescence micrographs of a ~2.5 cm long peripheral nerve stump (sciatic-tibial nerve segment) wholemount preparation at the proximal (A) and distal site (B) 37 h after cut injury with few individual fluorescent axons broken into fragments. A small number of axons fragmented at the proximal (C) and distal site (D) of a peripheral nerve stump wholemount preparation could also be detected 40 h following crush injury. **E-H**: Conventional fluorescence micrographs of a ~2.5 cm long peripheral nerve stump (sciatic-tibial nerve segment) wholemount preparation at the proximal (E) and distal site (F) 42 h after cut injury with most YFP labelled axons fragmented. A similar picture with a majority of axons degenerated is evident at the proximal (G) and distal end (H) of a peripheral nerve stump wholemount preparation 44 h after crush injury. YFP fluorescence has been pseudo-coloured green with the applied imaging software (MetaVue, Universal Imaging Corporation). Magnification: 100 ×

In preliminary experiments we used conventional light and electron microscopy to investigate whether Wallerian degeneration is progressive in wild-type mouse peripheral nerves. We were never able to find any significant gradients of degeneration along injured nerves that were processed with these traditional methods (data not shown). We then looked for signs of progression in localised observations of degenerating YFP-H nerves because fragmentation of YFP-labelled axons from these mice corresponds to granular disintegration of axoplasm as well as myelin ovoid formation and YFP positive axons represent the whole myelinated axon population [54]. Axonal fragmentation was first detected at both the proximal and distal ends of the distal nerve stump 37 h after transection (Fig. 1A, B) and 40 h after crush injury (Fig. 1C, D). 42 h after transection (Fig. 1E, F) and 44 h following crush lesion (Fig. 1G, H) the majority of axons in both locations were fragmented by assessment with conventional fluorescence microscopy. By direct comparison of the separate images of proximal and distal sites in the distal nerve stumps excised at all further time points no apparent difference was visible in the proportion of fragmented axons (data not shown).

### Wallerian degeneration in wild-type nerves progresses anterogradely after nerve transection and retrogradely after nerve crush

**Figure 2:**
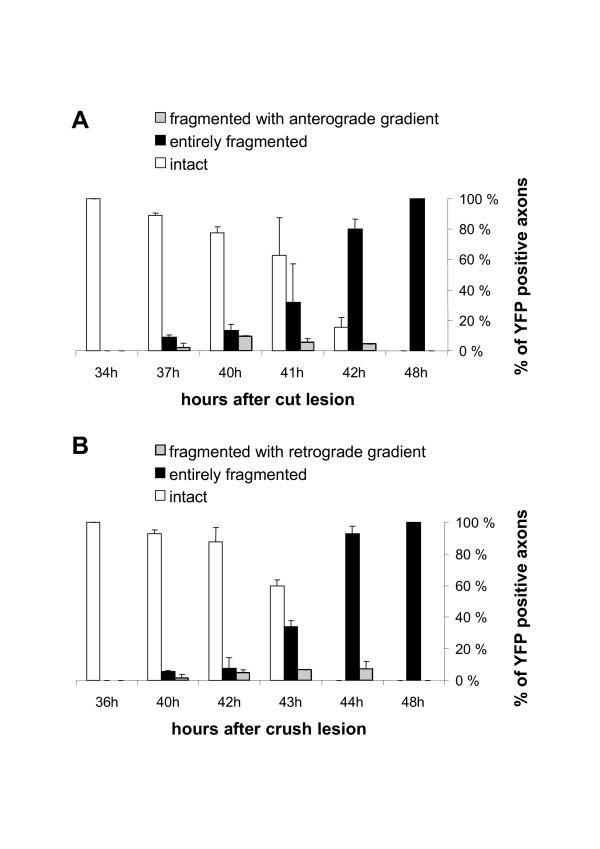
**Quantification of fluorescent axons in wholemount YFP-H peripheral nerve stumps after cut and crush injury at different time points. **Depending on the extent of fragmentation, YFP positive axons from peripheral nerve stumps were assigned to the group "intact", "entirely fragmented", "fragmented with anterograde gradient" or "fragmented with retrograde gradient". The chart presents means and standard deviations. **A**: All partially fragmented axons that could be identified at the time points between 37 h and 42 h after cut injury were fragmented at the proximal end of the distal axon stump but not further distal, indicating an anterograde gradient of Wallerian degeneration ("fragmented with anterograde gradient"). A maximum of 9.3 % YFP positive axons with anterograde fragmentation appeared 40 h after cut injury. **B**: All partially fragmented axons that could be identified at the time points between 40 h and 44 h after crush injury were fragmented at their distal ends but not further proximal indicating a retrograde gradient of Wallerian degeneration ("fragmented with retrograde gradient"). A maximum of 7.2 % of YFP positive axons with retrograde fragmentation appeared 44 h after crush injury.

**Figure 3:**
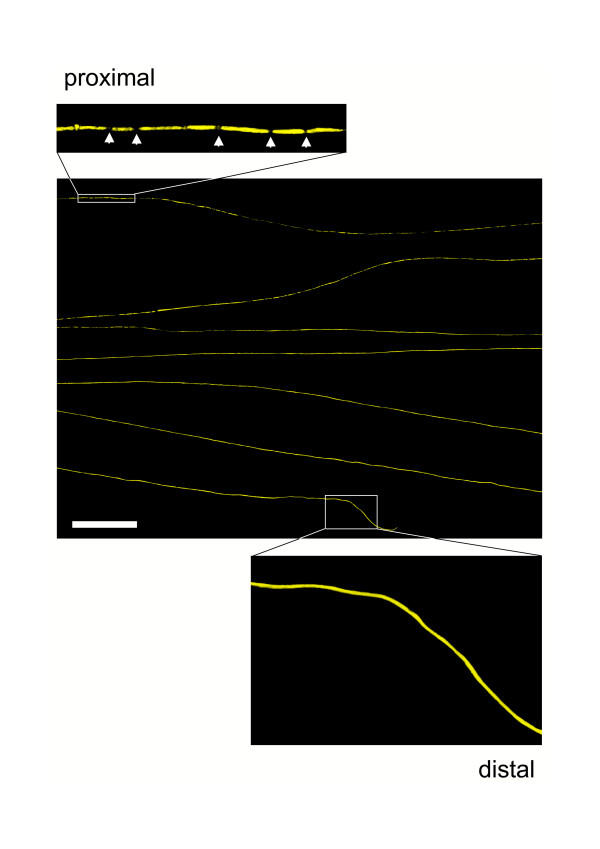
**Wallerian degeneration proceeds in anterograde direction along individual axons after cut injury. **Confocal composite picture showing seven consecutive lengths (from top to bottom in overview) of the proximodistal course of an individual YFP labelled axon within a distal nerve stump 40 h after transection demonstrating an anterograde progression of axon fragmentation. Note that this axon has fragmented in its proximal end (upper inset) but not in its distal end (lower inset). Axonal fragments are clearly demarcated by fluorescence interruptions (arrows in upper inset). YFP fluorescence has been pseudo-coloured yellow with the applied confocal imaging software (Biorad LaserSharp 2000). Scale bar: 500 μm

The failure to observe any gradient of degeneration in the above experiment does not mean that Wallerian degeneration is not progressive. It could propagate so rapidly that it was not detectable by this method, or a gradient might not be detectable because of the considerable statistical noise of the highly asynchronous process. In order to investigate these possibilities we turned to confocal tracing of individual axons in long wholemount YFP-H nerve segments. At 37 h after transection we found 2.0 % of fluorescent axons with extensive proximal fragmentation and intact distal regions indicating an anterograde gradient of Wallerian degeneration in these axons (Fig. 2A). Around 40 h the proportion of distal axon stumps degenerated at their proximal but not distal ends peaked at 9.3%. An example is presented in Fig. 3. Of the remaining axons, 77.5 % were intact and 13.2 % were entirely fragmented without an apparent gradient. 42 h after cut injury the proportion of partially fragmented axons decreased to 4.4 % and after 48 h we only found axons that were fragmented over the whole length. In summary, at all investigated time points partially degenerated axons (mean: 5.3 %) always exhibited an anterograde spread of Wallerian degeneration after nerve transection.

**Figure 4:**
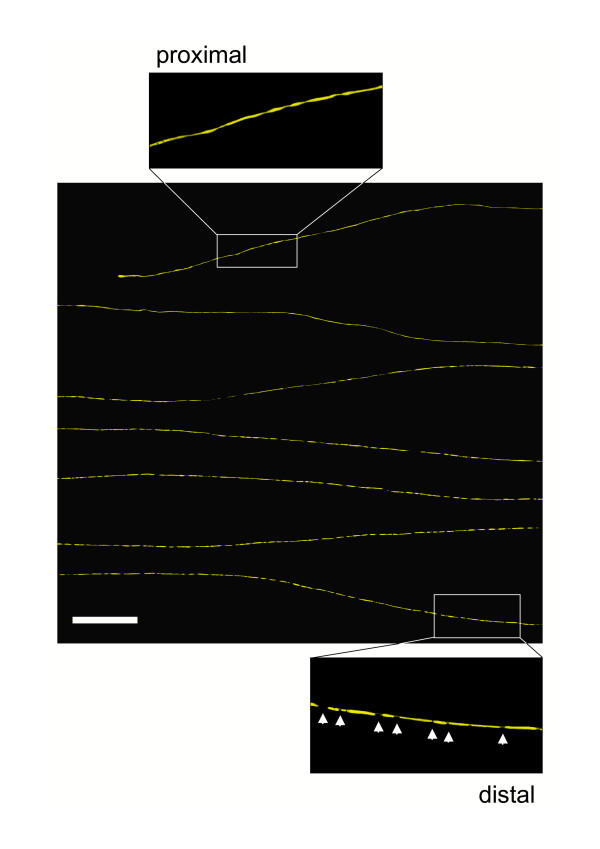
**Wallerian degeneration proceeds in retrograde direction along individual axons after crush injury. **Confocal composite picture showing seven consecutive lengths (from top to bottom in overview) of the proximodistal course of an individual YFP labelled axon within a peripheral nerve stump 44 h after crush injury displaying a retrograde progression of axon fragmentation. Note that this axon has fragmented in its distal end (lower inset) but not in its proximal end (upper inset). Axonal fragments are clearly demarcated by fluorescence interruptions (arrows in lower inset). YFP fluorescence has been pseudo-coloured yellow with the applied confocal imaging software (Biorad LaserSharp 2000). Scale bar: 500 μm

In contrast, all partially fragmented axons after crush injury at all investigated timepoints were fragmented in distal tibial nerve but not at the proximal end of the distal stump (Fig. 2B). Once again, only a small minority (mean: 5.0%) could be detected in this state at any one time. Partially fragmented axons first appeared at 40 h (1.6 % of axons) and the proportion peaked at 44 h (7.2 %). A representative example is shown in Fig. 4. The remaining 92.8 % of YFP labelled axons at 44 h was entirely fragmented without an apparent gradient. At all earlier investigated time points axons with a retrograde gradient of fragmentation were also observed but in lower proportions and after 48 h we only found axons that were fragmented over the whole length.

Summarising all these quantification results of cut and crush lesions at time points where partially fragmented fibres were observed, on average 94.8 % of all axons were either completely intact or fragmented and 5.2 % showed a gradient of Wallerian degeneration, whose orientation depended on the lesion type.

### A wave of axonal fragmentation propagates rapidly along individual wild-type axons and the axon population degenerates asynchronously

To study further the gradients of axonal fragmentation both after cut and crush injury (Fig. 3, 4) we quantified the number of axonal breaks along partially and totally fragmented axons. Firstly, this was a way to distinguish between a locally restricted wave of fragmentation such that entirely intact lengths of axon suddenly change into entirely fragmented lengths, and a more gradual fragmentation process that would result in a few interruptions that become more frequent further along the axon. Secondly, by this approach we tried to get insight on the question of whether axons assigned into the group "entirely fragmented" continue to break into smaller fragments leading to a gradient of fragment size along the nerve.

**Figure 5:**
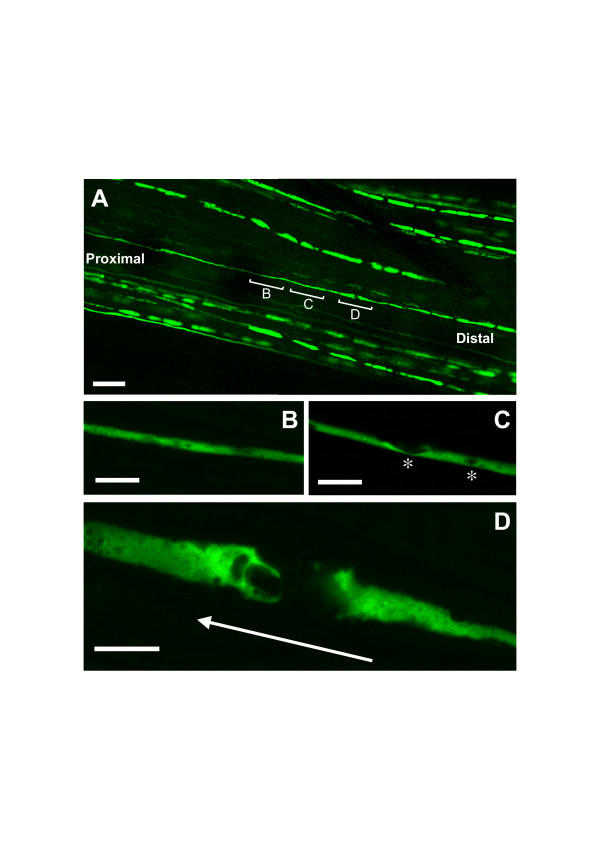
**Wave front of Wallerian degeneration in a YFP labelled wild-type axon after crush lesion A**: The partially degenerated axon that is bracketed was identified in a 44 h crushed wild-type nerve. All more distal regions of this axon are fragmented and all more proximal regions are intact (data not shown). **B-D**: higher magnification of this axon from (A) around the transition point between intact and fragmented regions. (D) shows the most proximal breakpoint in this nerve and the inferred retrograde direction of propagation of Wallerian degeneration. Immediately proximal to the breakpoint severe vacuolation occupies almost the entire axon thickness. Slightly further proximal in (C), there are also severe YFP negative vacuoles and fragmentation appears imminent at two points (asterisks). Further proximal still in (B), the degree of vacuolation decreases. YFP fluorescence has been pseudo-coloured green with the applied confocal imaging software (Zeiss LSM Software Release 3.2). Scale bars: 50 μm (A) and 10 μm (B, C, D)

**Figure 6:**
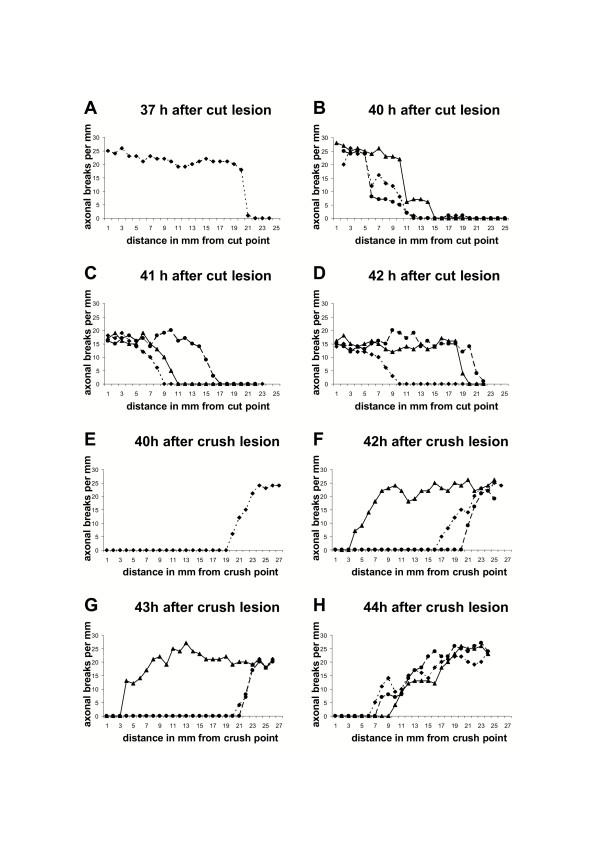
**Axonal fragmentation progresses asynchronously as a localised wave along individual axons in a anterograde or retrograde direction A-D**: Graphs showing the number of axonal breaks along individual YFP labelled axons with anterograde gradient of fragmentation in relation to the distance in mm from the transection point 37 h (A), 40 h (B), 41 h (C) and 42 h (D) after cut lesion. Note that with increasing distance from the transection, axon lengths with marked fragmentation abruptly change into lengths with no or just a few axonal breaks, indicating that Wallerian degeneration progresses with a localised fragmentation wave front. Additionally note the variable localisation of the fragmentation wave front along different axons at one timepoint representing the asynchronity of Wallerian degeneration among the axon population. **E-H**: Graphs showing the number of axonal breaks along individual YFP labelled axons with retrograde gradient of fragmentation in relation to the distance in mm from the crush point 40 h (E), 42 h (F), 43 h (G) and 44 h (H) after crush lesion. Note that with increasing distance from the crush point axon lengths without any features of fragmentation abruptly change into lengths containing axonal breaks. Asynchronity of progression of Wallerian degeneration along individual axons is also apparent after crush lesion.

Concerning the first question we found that Wallerian degeneration progresses as a wave, with the wave front defining the point to which fragmentation had spread along the axon. In partially fragmented axons separated from the cell body by transection or proximally compressed by crush lesion, axon regions with no features of degeneration abruptly change into segment lengths with marked breakdown within a transition zone of less than one millimeter (Fig. 5, 6). A short region of intact axon immediately ahead of the wavefront becomes increasingly vacuolated as the wave front approaches, and a newly formed break appears as though a vacuole has filled the entire axon diameter, completely interrupting it (Fig. 5). The degeneration wave sequentially affects adjacent regions of the fibre and different lesions cause this wave to progress in different directions.

In order to determine whether the anterograde and retrograde fragmentation wave runs at the same velocity along the axon we next estimated the rate of progression. As the average axon length measured was 24 mm and the majority of axons must have entirely fragmented between 41 h and 42 h after transection (significant difference between percentage of entirely degenerated axons at 42 h and entirely plus partially degenerated axons at 41 h in Student t-test) (Fig. 2A), the minimal velocity for the degeneration wave is 24 mm/h. Analogously, after crush lesion the calculated velocities of the retrograde degeneration wave is also at least 24 mm/h (Fig. 2B), as the majority of axons fragmented between 43 h and 44 h after the lesion (significant difference between entirely degenerated axons at 44 h and entirely plus partially degenerated axons at 43 h in Student t-test). Thus, the rates of Wallerian degeneration progression are similar or possibly even equal in these opposite directions, but with a faster initiation of the fragmentation wave after transection.

We then tested whether our crush lesions interrupted axon continuity as in nerve transection, because a failure to do so could underlie the different direction of propagation in crushed nerves (see Discussion). In fluorescent wholemount preparations of crushed nerve segments we found continuous longitudinal YFP signals across the crush site, and in teased fibre bundles of osmificated nerve segments after crush injury, a majority of fibres remained continuous across the crush site (see additional data file *Add Fig 1.pdf*). These data are consistent with the axonal membrane remaining intact after 30 sec nerve crush, unlike that of a transected nerve.

The observation that at early time points after cut and crush injury some axons had already fragmented or started to fragment while the majority is still intact (Fig. 1, 2) together with the variable localisation of the fragmentation wave front along different axons at one time point (Fig. 6) indicates that Wallerian degeneration is asynchronous among the population of axons in a peripheral nerve. This probably reflects both differences in the timing of onset of degeneration and varying velocities of propagation in axons of different thickness that cannot be distinguished by our imaging approaches. Furthermore, the observation that the transition between intact and degenerated regions can be 19–21 mm distal to the crush within 44 h rules out regeneration as a possible source of error.

**Figure 7:**
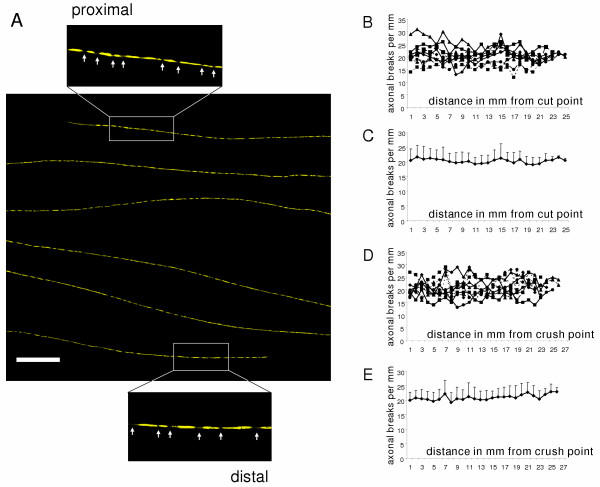
**The Wallerian degeneration wave runs through individual axons and leaves uniformly degenerated fibres without gradients of fragmentation A**: Confocal composite picture showing six consecutive lengths (from top to bottom in overview) of the proximodistal course of an individual completely fragmented YFP labelled axon within a peripheral nerve stump 42 h after transection injury without any features of a degeneration gradient. Note that this axon has fragmented in its proximal (upper inset) and distal (lower inset) site equally. Axonal fragments are clearly demarcated by fluorescence interruptions (arrows in insets). YFP fluorescence has been pseudo-coloured yellow with the applied confocal imaging software (Biorad LaserSharp 2000). Scale bar: 500 μm **B, C**: Graphs showing the number of axonal breaks along 10 YFP labelled axons without apparent gradient of fragmentation in relation to the distance in mm from the cut point 37 h to 42 h after cut lesion. Means and standard deviations are presented in (B). Note that axonal breaks and therefore fragmentation is homogenously dispersed through the axon lengths. **D, E**: Graphs showing the number of axonal breaks along 10 YFP labelled axons without apparent gradient of fragmentation in relation to the distance in mm from the crush point 40 h to 44 h after crush lesion. Means and standard deviations are presented in (E). Note that axonal breaks and therefore fragmentation is homogenously dispersed through the axon lengths.

**Figure 13:**
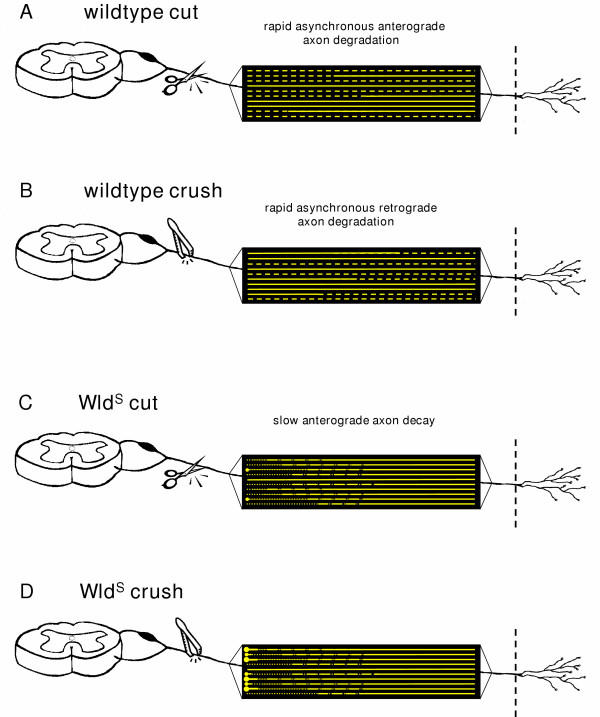
**Schematic illustration depicting the spatiotemporal pattern of axon degeneration after cut and crush injury of a wild-type and a Wld^S ^peripheral nerve. **Each yellow line represents an individual YFP positive axon in wild-type (A, B) and Wld^S ^(C, D) peripheral nerves. Accounting for wild-type peripheral nerves, firstly, both after transection (A) and crush injury (B) axonal fragmentation progresses as a localised wave quickly within a matter of few hours over the individual axon. Thereby, the abrupt shift between preserved and fragmented axon distances along partially fragmented axons represents the wave front. The processes differ only in direction with an anterograde course after cut and a retrograde course after crush lesion. Secondly, axonal fragmentation in the YFP positive axon population is asynchronous with some intact and others entirely or partially fragmented in one nerve at one time point. Thirdly, axonal breaks are dispersed homogenously along totally fragmented fibres. In contrast, in Wld^S ^peripheral nerves, firstly, both after transection (C) and crush (D) injury axonal degeneration progresses in anterograde direction with a velocity similar to that of slow axonal transport. Secondly, the gradients of axon degeneration are uniform with gradual decrease of degenerative changes along the axon from proximal to distal. Thirdly, degeneration happens broadly synchronously among the population of Wld^S ^axons. Fourthly, formation of end bulbs with subsequent swellings at the proximal ends of Wld^S ^axons can be observed especially after crush lesion but also occasionally after transection lesion.

Quantification of axonal breaks along entirely fragmented axons (Fig. 7) revealed that fragmentation is homogenously dispersed through the whole fibre distance and no gradient is detectable. Thus, once fragmentation begins it is rapidly completed. All these findings obtained in YFP-H mice are summarized schematically in Fig. 13.

### In contrast to wild-type nerves injured Wld^S ^sciatic and tibial nerves degenerate anterogradely independent of the lesion type

**Figure 8:**
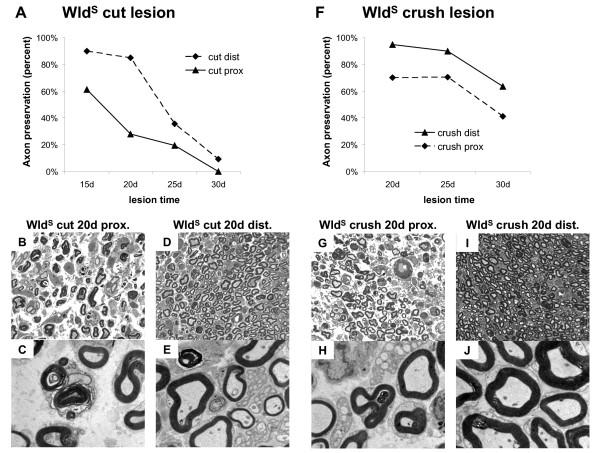
**Light and electron microscopy revealed an exclusively anterograde gradient of axon degeneration in transected and crushed Wld^S ^sciatic/tibial nerves after prolonged lesion times A, F**: Quantification of axon preservation at proximal and distal ends of the peripheral nerve stump after transection (A) and crush (F) injury exposed exclusively anterograde gradients of axon degeneration after 15 to 30 days following injury (15 d lesion time-point only after transection injury). Differences in the number of protected axons between the proximal and distal end of the stump were maximum after 20 days and more moderate prior or later to that, correspondingly. Remarkably, after 30 days following crush lesion considerable numbers of totally intact axons could be counted (63.5 % in distal tibial nerve) pointing to a weaker effect of compression over transection and generally to the longevity of distal Wld^S ^axons. **B-E**: Light microscopic images (B, D) and corresponding electron micrographs (C, E) taken from the proximal (B, C) and distal (D, E) end of the peripheral nerve stump after 20 days following transection lesion. At the proximal end (sciatic nerve) 28.1 % myelinated axons were structurally preserved while at the distal end (tibial nerve) we could observe 85.0 % preserved axons pointing to an anterograde gradient of axon degeneration. **G-J**: Light microscopic images (G, I) and corresponding electron micrographs (H, J) taken from the proximal (G, H) and distal (I, J) end of the peripheral nerve stump after 20 days following compression lesion. Similar to the transection lesion also here we identified a clear anterograde degeneration gradient with 70.0 % intact axons at the proximal end and 94.8 % preserved axons at the distal end of the nerve stump. Magnification of light microscopy is 630 × and electron microscopy is 3400 ×

We then extended these studies to Wld^S ^axons, already known to survive 14 days after transection lesion [13], using light and electron microscopy after prolonged lesion times of 15, 20, 25 and 30 days. In contrast to the analogous experiment in wild-type mice, a significant difference in axon preservation rate was immediately apparent between the proximal sciatic nerve and distal tibial nerve. 20 days after high sciatic nerve transection, 28.1 % of myelinated axons were structurally preserved a few millimetres distal to the lesion site in light and electron microscopy (Fig. 8A, B, C) but the most distal part of the tibial nerve showed ultrastructural preservation in 85.0 % of axons (Fig. 8A, D, E). Likewise, at all further time points beside 20 days (15, 25 and 30 days) after transection lesion we found more intact axons in distal tibial nerve than in proximal sciatic nerve close to the point of injury (Fig. 8A). Overall, these results clearly indicate anterograde progression of axon degeneration along transected Wld^S ^peripheral nerves.

Remarkably, in view of data reported in wild-type mice, a crush injury of the proximal sciatic nerve also resulted in anterograde progression in Wld^S ^sciatic/tibial nerve that was evident with a more moderate gradient after 20–30 days. Twenty days after sciatic nerve crush 70.0 % of all myelinated axons were preserved a few millimetres distal to the lesion (Fig. 8F, G, H) while 94.8 % of these fibres were preserved at the distal tibial nerve more than 20 millimetres away from the point of lesion (Fig. 8F, I, J). Similarly, 25 and 30 days after crush lesion we observed an increase in preserved axon numbers from proximal to distal along the sciatic-tibial nerve distance (Fig. 8F). Even 30 days after transection or crush lesion there were many protected distal axons in tibial nerve (Fig. 8A, F). By contrast, the degeneration of all distal fibres in wild-type mice was complete within two days (see above). Thus, while axon degeneration in more proximal regions is delayed approximately tenfold by Wld^S ^after a lesion, the delay in more distal regions is at least twenty fold. In summary, quantification of axon preservation assessed by light and electron microscopy is sufficient to indicate a marked anterograde direction of axon degeneration both after transection and crush injury of peripheral Wld^S ^nerves.

### Anterograde degeneration of individual YFP labelled Wld^S ^axons is slowly progressive

**Figure 9:**
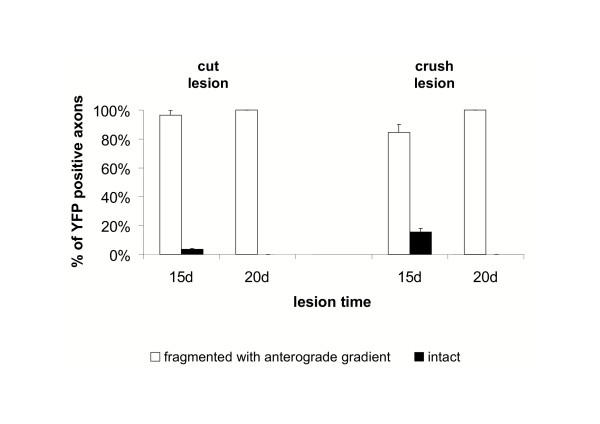
**Quantification of fluorescent Wld^S ^axons in whole-mounted peripheral nerve stumps from triple heterozygote mice after transection and crush injury at different time points. **Partially degenerated YFP positive Wld^S ^axons that could be identified 15 and 20 days either after transection or crush injury showed axonal constrictions or interruptions in their proximal site but not further distal indicating an anterograde gradient of degeneration. They were assigned to the group "fragmented with anterograde gradient". Few entirely preserved fluorescent Wld^S ^axons could be only seen 15 days after transection and crush injury. They were assigned to the group "intact". The chart presents means and standard deviations.

**Figure 10:**
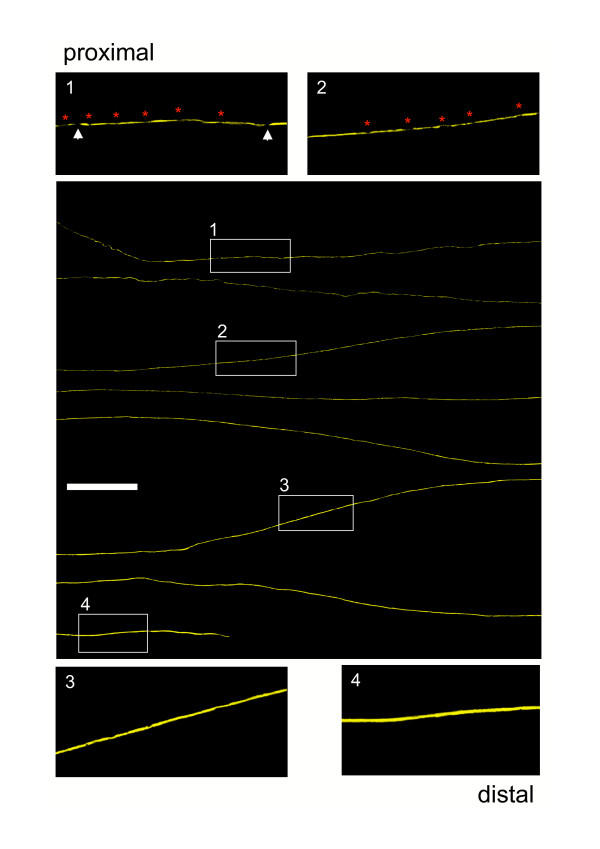
**Anterograde degeneration of transected Wld^S ^axons initially involves proximal axonal atrophy with occasional interruptions. **Confocal composite picture showing eight consecutive lengths (from top to bottom in overview) of the proximo-distal course of an individual YFP labelled Wld^S ^axon within a peripheral triple heterozygote nerve stump 15 days after transection injury displaying an anterograde progression of axon degeneration. Note that this axon shows predominantly narrowings (red asterisks) and occasionally interruptions (white arrows) in its most proximal end (inset 1) with a gradual decrease of this degeneration signs over a few millimetres more distal (inset 2) while at its distal parts almost no degeneration can be identified (inset 3 and 4). YFP fluorescence has been pseudo-coloured yellow with the applied confocal imaging software (Biorad LaserSharp 2000). Scale bar: 500 μm

**Figure 11:**
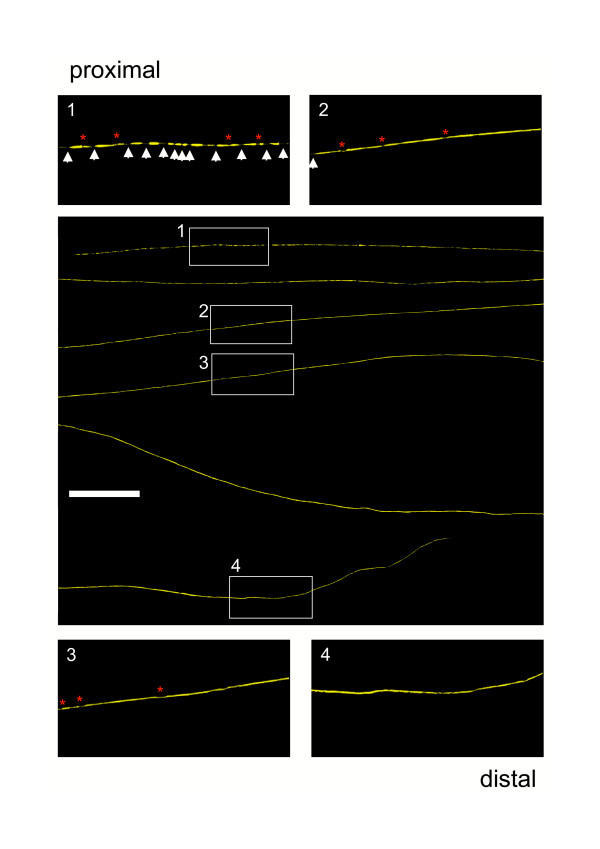
**Anterograde degeneration of transected Wld^S ^axons eventually continues with complete proximal fragmentation. **Confocal composite picture showing six consecutive lengths (from top to bottom in overview) of the proximo-distal course of an individual YFP labelled Wld^S ^axon within a peripheral triple heterozygote nerve stump 20 days after transection injury demonstrating a clearer anterograde progression of axon degeneration than in Fig. 10. Note that this isolated axon shows complete break-up (white arrows) with clearly demarcated fragments in its most proximal part among a minority of axonal narrowings (red asterisks) (inset 1). Moving further distal fragmentation accompanied by axonal constrictions becomes gradually weaker (inset 2, 3) while at its most distal end almost no degeneration can be identified (inset 4). YFP fluorescence has been pseudo-coloured yellow with the applied confocal imaging software (Biorad LaserSharp 2000). Scale bar: 500 μm

In order to exclude nerve branching as an explanation for the observations above, we carried out detailed longitudinal analysis of individual degenerating Wld^S ^axons labelled with the YFP-H transgene as described previously [54]. Following transection or crush lesions to the sciatic nerve, long-range confocal YFP axon tracing was performed in 2–3 cm wholemount nerve segments. 15 days after transection almost all (96.4 ± 3.4 %) YFP labelled Wld^S ^axons showed a homogeneous anterograde gradient of degenerative changes along the sciatic and tibial nerve (Fig. 9). An example is presented in Fig. 10. In contrast to partially degenerated wild-type axons after transection lesion, where there were clear interruptions between markedly demarcated YFP-positive fragments, axon fragmentation in proximal Wld^S ^nerves after 15 days was mostly incomplete. Instead of interruptions, there were many constrictions of short regions of the axon or thin axoplasmatic bridges between thicker regions (inset 1 + 2 in Fig. 10). Occasionally we observed small swellings (bulbs) at the proximal ends (data not shown). Distal areas of the same Wld^S ^axon lacked these degenerative changes (inset 3 + 4 in Fig. 10). The remaining 3.6 % of Wld^S ^axons appeared to be completely intact without any discernible signs of degeneration (Fig. 9). 20 days after transection, proximal regions of individual Wld^S ^distal axon stumps were more completely fragmented (inset 1 in Fig. 11). However, some millimetres distal the fragments became gradually less frequent and again were often joined by YFP positive material indicating incomplete fragmentation (inset 2 in Fig. 11). At this time point such incomplete fragmentation with axonal narrowing occasionally continued up to distal regions of the fluorescent Wld^S ^axons (inset 3 in Fig. 11). Altogether at 20 days post operation all axons showed anterograde gradients of complete or incomplete fragmentation (Fig. 9). Additionally, we analyzed individual axons that were separated for 12.5, 17.5 and 22.5 days from their parent cell body and found that with increasing lesion time proportionally more axons displayed anterograde degeneration gradients. The gradients became structurally clearer through more marked demarcation of axonal fragments (data not shown). Thus, the initial morphological events in the degeneration of Wld^S ^axons are constrictions or atrophy, followed only considerably later by complete interruptions of the axon.

We then followed up the earlier EM experiments using crushed nerves from Wld^S^/YFP-H double mutant mice to study the directionality of axon degeneration in Wld^S ^nerves using this very different method. Once again, the spatio-temporal pattern of axon degeneration after crush injury in Wld^S ^mice was very similar to that after transection injury, contrasting with wild-type mice where directionality depends on lesion type. Correspondingly, 15 days after crush lesion we counted 84.60 % of all YFP labelled Wld^S ^axons with an anterograde gradient of degenerative changes (Fig. 9). An example of such an individual axon is shown in the additional data file *Add Fig 2.pdf*. However, crushed Wld^S ^axons more frequently showed end bulbs at the proximal end of the distal stump, which were often very large, (red arrow in overview of *Add Fig 2.pdf*) and subsequent multiple axonal swellings (inset 1 in *Add Fig 2.pdf*). This feature was far more prominent in crushed Wld^S ^axons than in the transection experiment where we observed end bulbs just occasionally. Further distally these swellings disappear with remaining axonal constrictions and breaks (inset 2 in *Add Fig 2.pdf*) representing incomplete fragmentation. As in transected nerves, distal parts of the crushed Wld^S ^axon were free of degeneration signs (inset 3 and 4 in *Add Fig 2.pdf*). Compared to transection lesion, more axons at 15 days remained entirely intact (15.4 %) (Fig. 9). By 20 days after nerve crush of Wld^S ^axons, proximal fragmentation became more prominent with fully separated fragments (inset 1 in additional data file *Add Fig 3.pdf*) while more distal regions of the same axons were again incompletely fragmented (inset 2 in *Add Fig 3.pdf*) and further distal still lacked any degeneration signs (insets 3 + 4 in *Add Fig 3.pdf*). The proximal end bulbs and localised swellings were larger than at 15 days (red arrow in overview of *Add Fig 3.pdf*), possibly due to continued accumulation of retrogradely transported material.

In summary, quantification 20 days after crush lesion revealed that all axons showed anterograde gradients of complete or incomplete fragmentation (Fig. 9). Thus, the YFP-H studies confirmed our light and electron microscopy observations that delayed degeneration in individual Wld^S ^axons is directional with an exclusively anterograde pattern both after transection and crush injury. This pattern of degeneration is qualitatively different from that in wild-type mice, which shows asynchronous, bidirectional fragmentation and degeneration.

### Wld^S ^axons show a continuous gradient of axon degeneration that moves with a velocity similar to that of slow axonal transport

**Figure 12:**
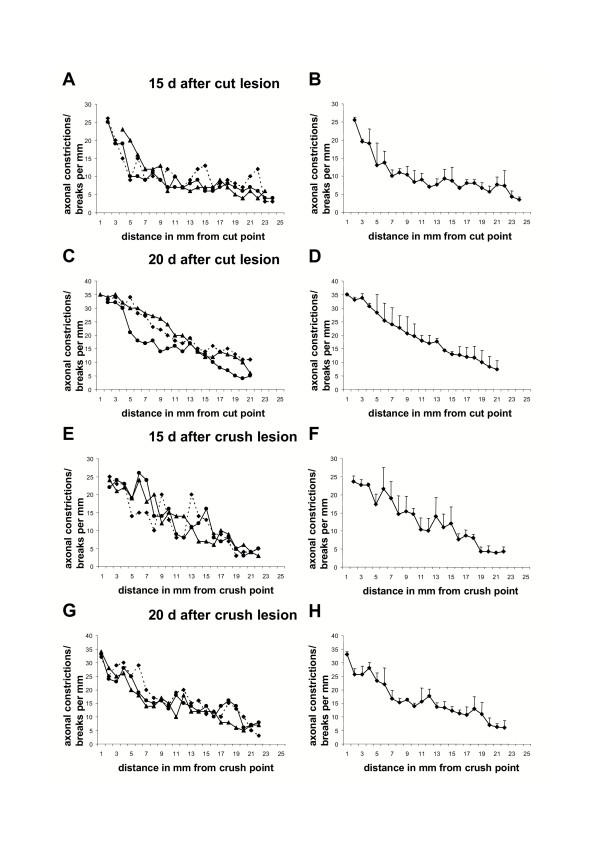
**Progression of axon degeneration in shape of a continuous degeneration gradient appears roughly synchronous along individual Wld^S ^axons A-H**: Graphs showing the number of axonal constrictions and breaks along individual YFP labelled Wld^S ^axons with an anterograde gradient of degeneration in relation to the distance in mm from the transection point 15 days (A, B) and 20 days (C, D) after transection lesion or from the crush point 15 days (E, F) and 20 days (G, H) after crush lesion. Means and standard deviations are presented in B, D, F, H. Note that with increasing distance from the transection and crush point degeneration signs decrease uniformly characterized by the steady decline of the curves. Moreover, degeneration in different Wld^S ^fibres is broadly synchronous as shown by the good superimposition of individual curves in A, C, E, G.

In the above experiments we noted the gradual change from degenerated regions to intact regions in Wld^S ^axons. In contrast to our observations in wild-type mice, there was no clearly delineated boundary or wave front separating degenerated and fully intact regions. In order to quantify this, we counted the number of axonal constrictions and breaks along the length of YFP positive Wld^S ^axons following injury. At all post-lesion time-points randomly chosen axons showed gradually decreasing signs of degeneration (constrictions or interruptions) along their length (Fig. 12). This markedly contrasts with the wave-like degeneration observed in wild-type mice where a sharp boundary divided preserved regions of the axon from completely fragmented regions. The closely superimposed curves shown in Fig. 12A, C, E, G also indicate that anterograde axon degeneration is more synchronous among the axon population in Wld^S ^nerves at both 15 and 20 days following transection or crush injury. Only rarely could we observe morphologically normal Wld^S ^axons adjacent to axons with anterograde gradients of degeneration (Fig. 9). This quantification allowed us to estimate the rate at which anterograde degeneration progresses along single Wld^S ^axons. Between 15 and 20 days the equivalent stage of degeneration has advanced up to 11 mm further along the nerve (sometimes less), giving a maximum velocity of Wld^S ^degeneration progression of 11 mm / 5 days = 2.2 mm/day. This is similar to the reported velocity of slow axonal transport (0.1–3.0 mm/day) [56-59]. All findings concerning topology of axonal degeneration in Wld^S ^peripheral nerves are summarized schematically in Fig. 13.

## Discussion

We have shown that the fragmentation of axons undergoing Wallerian degeneration in a mixed wild-type peripheral nerve is a rapid, asynchronous and progressive process. By using a recently developed method to visualise individual axons over cm-long distances, and by targeting a short critical period during which nearly all axons degenerate, we have made the first observations in vivo of partially fragmented individual axons and thus determined the directionality and the wave-like nature of Wallerian degeneration, as well as estimating its velocity. Furthermore, we have shown that nerves of Wld^S ^mutant mice undergo a fundamentally distinct process rather than simply following the same pathway in slow motion.

Lesioned wild-type axons remain morphologically normal for a latency period of ca. 36–44 hours, which depends on lesion type and individual axonal properties [38,39,44,46,60]. Each axon then undergoes a catastrophic process in which at least 24 mm of the distal stump fragments entirely within an hour. The propagation rate of at least 24 mm/h is considerably faster than reported in rat dorsal column (3 mm/h) [47], rat phrenic nerve (up to 10.4 mm/h) [46] and in primary culture (ca. 0.4 mm/h) [50], probably reflecting differences in neuronal subtype and context. For example, slower propagation of Wallerian degeneration in the CNS is suggested by the observation of an anterograde spread in the gracile tract following a dorsal root lesion, whereas the spread of degeneration within the root itself was too short-lived to be resolved by the methods used [47]. Wallerian degeneration may also propagate more slowly in longer axons, which could account for differences between mice and rats [44,45], and there may be many reasons why the propagation rate in vitro could differ from that in vivo. Nevertheless, while the propagation rate may differ, the anterograde degeneration after axon transection is a consistent feature of each of these studies.

We have shown that Wallerian degeneration in wild-type nerves is a wave-like process that can travel in either direction along the axon, depending on lesion type. 29 partially fragmented axons were observed, and all showed a sharp boundary between fragmented and non-fragmented zones, such that all axon regions up to the wavefront were degenerated and all regions beyond it remained intact. Fragmentation had reached different points along the nerve in different individual axons (Fig. 6), reflecting asynchronicity of the onset and rate of degeneration. The wave-like propagation of Wallerian degeneration has been proposed before [44,46,47,49,61], and especially Lubinska [44,46] has shown that Wallerian degeneration of the distal stump progresses centrifugally by jumping from one internode to another, but this is the first time the wavefront has actually been observed. Of the 29 partially fragmented axons, 17 transected axons were fragmented only at their proximal ends and 12 crushed axons were fragmented only at their distal ends (Fig. 2, 3, 4). Differences between cut and crushed nerves have been suggested before [48,53] and axons cut at both ends also exhibit a retrograde degeneration component [44], but this is the first demonstration that two different lesions at the same site in the same nerve cause different directions of degeneration. The mechanistic basis of this surprising observation remains unknown, but some models are outlined below.

**Figure 14:**
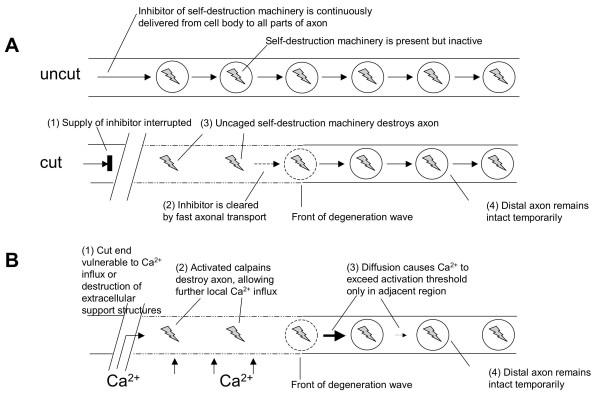
**Two models to account for the progressive nature of Wallerian degeneration after transection lesions in wild-type axons. **(A) A putative inhibitor of intrinsic self-destruction machinery is constantly delivered from the cell body to the unlesioned wild-type axon (top). After axon transection the inhibitor is no longer supplied and is cleared first from proximal regions of the distal stump by fast axonal transport. This leads to a wave of fragmentation moving proximal to distal along the isolated axon stump. (B) In an alternative model, the wave of fragmentation is propagated not by directional removal of a putative inhibitor but by rapid localised influx of calcium ions beginning at the most vulnerable part of the axon. Once inside, calcium ions not only activate calpains to degrade the local axoplasm, but also diffuse and exceed the threshold of calpain activation in the immediately adjacent region. This leads to further axoplasmic and membrane breakdown and further calcium influx. The pattern is repeated to generate a wave of fragmentation moving along the axon. Model (A) has the attraction that the putative inhibitor would be a good candidate for mediating of the Wld^S ^phenotype (e.g., it could be overexpressed in Wld^S^), while model (B) more easily explains why the directionality is reversed in a crush lesion. The calcium influx and diffusion wave could spread also retrogradely (not shown) if the distal end were the first to disintegrate. In model (A), however, it is hard to see how retrograde axonal transport could explain the depletion of an inhibitor that ultimately has to come from the cell body (see text for more details).

We discuss here two models to explain the wave-like propagation of Wallerian degeneration in wild-type nerves: one based on fast axonal transport and the other based on calcium influx (see also Fig. 14). Numerous reports have proposed that the clearance of a supportive or trophic factor by fast axonal transport processes underlies the anterograde direction of Wallerian degeneration after transection [33,44,46,47,62] based on the observation that anterograde fast axonal transport of proteins continues after axotomy in the peripheral nerve stump in a wavelike manner. Such a factor could be an inhibitor of an axonal destruction programme, likely to be stabilised or upregulated by a downstream effector of the Wld^S ^protein. The fastest reported components of axonal transport move at around 14–25 mm/h, but it is reasonable to expect that some minor, thus far undetected, components may move faster [63,64]. This is just compatible with the spread of the fragmentation wave at a minimum of 24 mm/h that we observe. However, Wallerian degeneration could progress even faster, too fast to be accounted for by fast axonal transport, and anterograde clearance of a factor inhibiting Wallerian degeneration could not explain the retrograde degeneration that we found in crushed nerves.

Thus, we consider also an alternative model in which short regions of the axon membrane become permeabilised to calcium ions and this feature moves rapidly as a wave along the axon. Fibre degeneration requires accumulation of axoplasmic calcium [65,66], which probably activates the cystein protease calpain [67-70]. Once inside the axon, calcium ions could diffuse to the immediately neighbouring axoplasm and activate calpain, leading to degradation of axoplasmic and membrane proteins, and thus permeabilisation of the next segment. Such a membrane associated Ca^2+ ^influx hypothesis was proposed by Schlaepfer for the first time [71,72] and developed further involving calcium channels in more modern studies [73,74]. LoPachin and Lehning [75] reported calcium entry linked to membrane depolarisation through reverse Na^2+^-Ca^2+^-exchange, leading to a steady rise in intra-axonal calcium and calcium accumulation has been observed beneath Schmidt Lantermann clefts at distal sites 4 h after injury [76]. Once the threshold for calpain activation is exceeded, a wave of degeneration could be initiated and then propagate rapidly in either direction as outlined above.

There are several possible reasons why degeneration may begin proximally in a transected nerve but distally in a crushed nerve. The proximal end of a transected distal stump is especially vulnerable because of the exposure of the axoplasm to the external ionic environment, and because all extra-axonal structures that normally support the axon have been totally disrupted, e.g., blood vessels, Schwann cells, extracellular matrix, perineurium. One of these factors may cause a calcium entry wave to begin at this point. In contrast, intact endoneural blood vessels can be found close to a nerve crush [77,78], the epi- and perineurium tubes are maintained at the site of crush [78-81], and some nerve crush protocols do not break axon continuity [82-85], so that Wallerian or Wallerian-like degeneration occurred only many days after compression or not at all. More specifically, even application of longer high pressure injuries with a minimum of shear forces may squeeze out axoplasm into adjacent parts of the axon rather than interrupting the axolemma preserving nerve conduction monitored electrophysiologically [82]. In our nerves we observed continuous longitudinal YFP signals across crush sites immediately after lesioning, indicating that at least some axons were not transected by the direct effect of crushing. We also observed many preserved fibres crossing the crush site when we fixed and then partially teased crushed nerves to generate small bundles where individual fibres were easily identifiable (see additional data file *Add Fig 1. pdf*). Thus, the proximal end of the distal stump may be less vulnerable than after transection, and fragmentation may begin instead at the distal end because this is the hardest part to supply with everything the axon needs to survive.

We observed a series of differences in the pattern of Wallerian degeneration in Wld^S ^nerves that are incompatible with delayed axon degeneration following a similar mechanism to Wallerian degeneration in wild-type nerves, only slower. The spread of degeneration along Wld^S ^nerves is around 100-fold slower, axon degeneration is more synchronous, at least relative to how long it takes to occur, it progresses in a proximal to distal direction in crushed nerves as well as transected nerves, there is a continuous gradient of degeneration along the length of the axon rather than an abrupt change at a boundary, and the first sign of axon degeneration is a constriction rather than a complete interruption. We therefore propose that the ultimate degeneration of axons in Wld^S ^mice be termed "slow anterograde axon decay" rather than Wallerian degeneration as summarised schematically in Fig. 13.

Based on these differences, we propose that injured Wld^S ^axons eventually undergo a passive process of atrophy, rather than an active process of self-destruction similar to apoptosis that appears to take place in wild-type axons [5,7,15,16,86]. It is likely that preserved axonal proteins will eventually be degraded by catabolic processes and may not be replaced by significant new synthesis, even if Wallerian degeneration is completely prevented. A direct indication of this is our observation in primary neuronal cultures of significant atrophy of distal neurites when their degeneration is delayed by Wld^S ^(data not shown). Even the fact that some proteins are synthesised locally in axons [57,87-89] may not be sufficient to prevent the eventual depletion of protein in severed Wld^S ^axons, as it remains unclear which proteins are made there and in what quantities.

We discuss two models to explain the slow anterograde progression of degeneration along Wld^S ^axons: one based on slow axonal transport and the other on a temperature gradient along the limb. The gradual nature of axonal atrophy in Wld^S ^makes it difficult to be precise about the rate at which it progresses along the axon, but it is certainly not incompatible with the velocity of slow axonal transport of 0.1–3.0 mm/day [56-58,90]. Clearance of structural proteins by slow anterograde transport, added to their gradual depletion by protein turnover, could cause the protein content at the proximal end of the distal stump to drop below the threshold level needed to maintain axon integrity. Bidirectional transport of cytoskeletal components continues in transected Wld^S ^nerves, leading to localised neurofilament-depleted constrictions and terminal and intermediate swellings containing disorganised neurofilaments [91,92]. We have made similar observations in YFP labelled Wld^S ^axons, and additionally report a gradient of such features along the nerve. A net anterograde movement of cytoskeletal proteins could therefore underlie the anterograde gradient of axonal atrophy in Wld^S ^axons injured for many days.

Alternatively, a proximal-distal decreasing gradient of temperature along the limb could underlie the observed difference in degeneration rate at different points in the Wld^S ^nerve. A decrease in temperature has been shown to delay degeneration both in wild-type and Wld^S ^axons after injury [52,74,93-96]. In wild-type nerves, a temperature gradient explains neither the different directions of propagation after transection and crush injury, nor the sharp boundary between intact and degenerated regions. However, in Wld^S ^axons there is a proximal to distal gradient of degeneration regardless of lesion type and the change from intact to degenerated is a gradual one. Thus, a temperature gradient could play a more important role here.

The extremely long survival of distal tibial nerve following injury in Wld^S ^is in marked contrast to the presynaptic nerve terminal at the neuromuscular junction, which is the first structure to degenerate in both wild-type and Wld^S ^nerves [2,8,46]. Intramuscular nerve also degenerates early, at least in Wld^S ^heterozygotes (L. Fan and R.R. Ribchester, unpublished). This supports the hypothesis of compartmentalised degeneration mechanisms of axons and synapses [97] and suggests that a clear boundary exists between the two domains. The location and nature of this boundary could hold important clues for determining the mechanism of both Wallerian degeneration and synapse degeneration.

Finally, the methods we report here could now be applied to study spontaneous nerve degeneration in 'dying-back' disease. The 'dying-back' model also predicts the transitory existence of partially degenerated axons, but as in Wallerian degeneration such axons have never been directly observed and there is no indication of the speed of 'dying-back' of individual axons [98,99]. There are interesting parallels between axon degeneration after nerve crush and 'dying-back', as both can be delayed by the *Wld*^*S *^gene and the direction of degeneration is also shared [24]. Thus, it is an intriguing possibility that the speed of propagation is equally rapid and asynchronous. If it is a similarly catastrophic event, what can stop it progressing back to the cell body leading to neuron death? In some cases neuronal death does appear to be the outcome [25,100], whereas in others, proximal axons and their cell bodies somehow survive [101] and it is important to find out why. Future prospects include direct observation of the progression of a degeneration boundary along YFP labelled axons *in vivo *after nerve lesion or in disease, once methods for *in vivo *imaging of single axons become more refined and more readily available, e.g., Cell ViZio, Mauna Kea Technologies [102].

## Conclusions

In summary, we report the first direct observation of partially degenerated single axons in lesioned nerves undergoing Wallerian degeneration, indicating that Wallerian degeneration propagates in wild-type nerves as a wave whose speed is at least as fast as the highest reported rate of fast axonal transport. It could be faster still in mouse sciatic and tibial nerve. The direction of degeneration is proximal to distal after a cut, but the reverse after a crush. For now the mechanism remains unknown, but these observations will ultimately need to be explained in any comprehensive model of the Wallerian degeneration mechanism. Injury-induced axon degeneration in Wld^S ^nerves is also progressive, but differences in the topographic pattern and morphology of degeneration indicate a fundamentally different process from that in wild-type nerves. We propose that Wld^S ^axons ultimately undergo atrophy, in a passive process similar to that which Wallerian degeneration was once thought to be.

## Methods

### Crossbreeding and genotyping of transgenic mice

Crossbreeding and genotyping of the YFP-H line [55] and triple heterozygote mice carrying the original *Wld*^*S *^mutation, the transgenic *Wld*^*S *^mutation (tg-*Wld*^*S*^) [13] and the YFP-H gene was performed as previously described [54]. Triple heterozygote mice rather than Wld^S^/YFP-H mice were used purely for reasons of convenience in their breeding. They express similar levels of Wld^S ^protein as homozygous natural mutant Wld^S ^mice and display a similar retarded time-course of axon degeneration. They additionally express YFP in approximately 3% of myelinated motor and sensory fibres in the PNS, equal to the original YFP-H line obtained from the Jackson Laboratories.

### Sciatic nerve lesions

Six- to 10-week-old mice from the YFP-H line and triple heterozygote mice for the second part of the study were anaesthetised by intraperitoneal injection of Ketanest (5 mg/kg; Parke Davis) and Rompun (100 mg/kg; Bayer), sciatic nerves were transected or crushed firmly close to the Foramen intrapiriforme and the wound was closed with a single suture. Complete nerve transection was performed with conventional surgical scissors and crush lesion was achieved with fine watchmaker's forceps (model: micrscopic forceps bent No. 7, Aesculap BD 333R, Germany) for 30 seconds. The continuity of the sciatic nerve was always preserved after crush lesion as checked in situ. For light and electron microscopy we removed distal ~2.5 centimeter long nerve stumps after 15 (transection only), 20, 25 and 30 days in triple heterozygote mice following intracardial perfusion. The first 2 millimeters of the distal nerve stump were discarded to reduce artifacts, and the next 2 mm from the most proximal and distal end of the peripheral nerve stump was prepared for Durcupan embedding and electron microscopy.

For conventional fluorescence microscopy and confocal tracing of individual YFP labelled wild-type axons after 34 h, 37 h, 40 h, 41 h, 42 h and 48 h following cut lesion and 37 h, 40 h, 42 h, 43 h, 44 h and 48 h following crush lesion the operated YFP-H mice were sacrificed by cervical dislocation and nerve segments prepared as follows.

For confocal tracing of individual YFP labelled Wld^S ^axons from triple heterozygote mice we dissected sciatic-tibial nerve segments after 12.5 d (transection only), 15d, 17.5 d (transection only), 20 d and 22.5 d (transection only). For each investigated time-point 2 – 3 YFP-H or triple heterozygote mice were operated.

### Assessment of axonal continuity in crushed YFP-H nerve segments

Sciatic nerves of mice from the YFP-H line were crushed as described in the paragraph above and the short nerve segment containing the crush site immediately removed after lesion, freed from surrounding connective tissue and subsequently either prepared for wholemount fluorescence embedding as described previously [54] or for tissue osmification. For the latter the crushed segment was immersion-fixed for four hours in 10% PFA in 0.1 M PBS, washed three times in 0.1 M PBS for 10 minutes and osmificated in aqueous osmium-tetroxide (1 %) solution for 90 minutes. After rinsing in fresh 0.1 M PBS individual axon bundles were teased from the crushed segment using fine syringe needles (Neoject 26 G × 1/2, Dispomed WITT, Germany) and mounted on conventional glass slides. Fluorescence imaging of crushed YFP-H nerve segments and light microscopy of osmificated fibre bundles was carried out using an Olympus IX 81 inverted microscope coupled to a Olympus U-TV0.5XC digital camera system.

### Intracardial perfusion for light and electron microscopy of semithin and ultrathin preparations

After sternotomy under deep anaesthesia mice were killed by cardiac puncture and instantly intracardially perfused first with a solution containing 10 000 i.e./l heparin (Liquemin N 25 000, Hoffmann-La Roche) and 1 % procainhydrochloride in 0.1 M PBS for 30 s and then with fresh half-strength Karnovsky's fixative (4 % paraformaldehyde, 2 % glutardialdehyde in 0.1 M sodium cacodylate, pH 7.3).

### Light and electron microscopy

Nerve samples for light and electron microscopy were embedded in Durcupan and further processed for examination with a Zeiss Axiophot light microscope and Zeiss EM 902 electron microscope as described previously [54].

### Morphological quantification of axon preservation in light and electron micrographs

The percentage of preserved myelinated axons in proximal and distal ends of peripheral nerve stumps from triple heterozygote mice after transection and crush injury was determined as described previously [13,54].

### Conventional fluorescence microscopy and confocal tracing of individual YFP labelled axons after various lesion times

In both YFP-H and triple heterozygote mice after cut or crush lesion the entire nerve distances from the proximal sciatic to the distal tibial nerve were carefully excised, the perineurium and the branch of the commune fibular nerve removed and the remaining ~2.5 centimeter long stumps treated for wholemount fluorescence preparation using Vectashield Mounting Medium (Vector Laboratories) as described previously [54]. For rough orientation the wholemount preparations were photomicrographed at the proximal and distal end of the excised nerve segment using a Zeiss Axiophot microscope connected to a digital camera system (Universal Imaging Corporation). High resolution confocal composite presentation of the entire intra-nerve course of individual degenerating YFP-labelled wild-type or Wld^S ^axons running through the excised stumps over their whole length was achieved as described previously [54]. Single images were obtained with LaserSharp 2000 software connected to a Biorad Radiance 2000 laser scanning system (Hemel Hempsted, UK) and composite pictures on a black background were created using Adobe Photoshop.

Imaging of restricted transition zones dividing intact and fragmented axon regions in YFP-H nerves was performed under highest possible resolution with a Zeiss LSM 510 META confocal system (LSM Software Release 3.2) coupled to a Zeiss Axiovert 200 microscope.

### Quantification of intact and degenerated YFP labelled wild-type and Wld^S ^axons in peripheral nerve stumps after cut and crush injury

For the first part of the study focusing on sciatic/tibial nerves from the YFP-H line in each wholemount preparation (2 – 3 nerve preparations per time point) YFP labelled wild-type axons running continuously through the excised peripheral nerve stump after cut and crush injury were traced individually with the laser scanning confocal microscope under high resolution and divided into four groups: axons without any features of axonal disintegration were assigned to the group "intact". Axons that showed fragmentation of the longitudinal YFP signal over the whole length of the fibre were assigned to the group "entirely fragmented". Axons without any sign of fragmentation at one end but clear axon breakdown at the other end were assigned either into the group "fragmented with anterograde gradient" or "fragmented with retrograde gradient" depending whether the fragmentation appeared close to the lesion point or at the most distal point of the tibial nerve segment. For the second part of the study dealing with triple heterozygote mice YFP labelled Wld^S ^axons in wholemount peripheral nerve stumps (2–3 nerve preparations for each timepoint) after transection and crush injury were equally traced individually with the BioRad Radiance 2000 laser scanning confocal microscope under high resolution, and divided into two groups: axons without any features of axonal disintegration were counted and assigned to the group "intact". Fibres that showed degeneration signs like constrictions or interruptions of the longitudinal YFP signal at the proximal site were associated into the group "fragmented with anterograde gradient". Means and standard deviations for all experiments were calculated using Microsoft Excel.

### Quantification of axonal degeneration signs along YFP positive axons

The length of degenerated YFP labelled wild-type and Wld^S ^axons was measured during the tracing process with the confocal laser scanning microscope and the distribution of axonal interruptions (both in wild-type and Wld^S ^axons) and/or constrictions (only in Wld^S ^axons) was graphed against the distance in mm from the cut or crush point. Means and standard deviations were calculated using Microsoft Excel.

### Animal experiments

All animal experiments were carried out under appropriate German licences: Tierschutzgenehmigung K 13, 11/00 and Anzeige K30/99.

## Authors' contributions

MPC (corresponding author) and BB jointly conceived of the design for this study, interpreted the experimental results and wrote the manuscript; BB carried out preliminary light and electron microscopy experiments, confocal imaging of YFP labelled axons, axon quantification and statistical analysis; RA: collaborated on the set up of the experiments, improvement of histological techniques and interpretation of results, performed teased fibre experiments, assisted in writing the manuscript; DW and DSG: provided excellent technical assistance in many parts of the experiments, performed sciatic nerve cut lesions and carried out routine work; KA: contributed to the discussion of the experimental results, preliminary light and electron microscopy work was done in his lab; RRR: contributed to the interpretation of the experiments and writing of the manuscript; confocal microscopy of YFP axons was mostly done in his lab; MPC: performed sciatic nerve crush lesions and partially confocal imaging, supervised all aspects of the study and main work was done in his lab.

All authors read and approved the final manuscript.

## Supplementary Material

Additional File 1 **Many nerve fibres are unbroken directly after a 30 second crush as shown in YFP-H wholemount nerves and osmificated teased fibre preparations.A-C**: Sciatic nerves crushed for 30 seconds at maximum pressure and then immediately fixed and imaged by osmium staining (A, B) or YFP fluorescence (C). **D: **YFP-H nerve crushed for 5 seconds at maximum pressure. Nerves in (A) and (B) were partially teased apart after staining, leading to accidental breakage of a few fibres. However, the majority of fibres clearly cross the crush site unbroken. YFP signal (identifying the axoplasm) at the crush site in (C-D) is weak, probably due to squeezing of the axoplasm longitudinally out of the crush site, or quenching of fluorescence by the crushed tissue, or both. To compensate, the photographs are deliberately overexposed. There is no sign of YFP positive axoplasm escaping laterally into the extracellular space, as would be expected if the axolemma were broken. Instead, some YFP positive axons clearly cross the crush site unbroken. In (C) the overexposure would prevent any broken axons from being identified, but in (D) the signal from most or all axons fades gradually as the axon enters the crush site, rather than stopping abruptly as one would expect if the axon were broken. Scale bars: 20 μm (A, B) and 50 μm (C, D). Click here for file

Additional File 2 **Degeneration of individual crushed Wld^S ^axons proceeds anterogradely beginning with the formation of end bulbs and axonal swellings at the most proximal end and accompanying proximal axonal atrophy.** Confocal composite picture showing seven consecutive lengths (from top to bottom in overview) of the proximo-distal course of an individual YFP labelled Wld^S ^axon within a peripheral triple heterozygote nerve stump 15 days after crush injury displaying an anterograde gradient of axon degeneration. This axon exhibits an end bulb at the most proximal end (red arrow in overview) and subsequent multiple axonal swellings delimited by constrictions (red asterisks) (inset 1). Further distally these swellings disappear with remaining constrictions (red asterisks) and sporadic breaks (white arrow) (inset 2). Distal parts of the crushed Wld^S ^axon are free of degeneration signs (inset 3 and 4). Scale bar: YFP fluorescence has been pseudo-coloured yellow with the applied confocal imaging software (Biorad LaserSharp 2000). 500 μm Click here for file

Additional File 3 **Anterograde degeneration with formation of massive end bulbs of compressed Wld^S ^axons finally includes complete proximal fragmentation.** Confocal composite picture showing six consecutive lengths (from top to bottom in overview) of the proximo-distal course of an individual YFP labelled Wld^S ^axon within a peripheral triple heterozygote nerve stump 20 days after crush injury demonstrating a clearer anterograde progression of axon degeneration than in *Add Fig 2.pdf*. Likewise after 15 days following crush injury this picture shows a massive end bulb at the most proximal end of the distal stump (red arrow in overview) and subsequent multiple axonal swellings and constrictions. Complete axonal fragmentation is evident in inset 1 (white arrows mark axonal breaks) and gradually gives way to incomplete breakup with axonal constrictions (red asterisks) (inset 2). Distal parts of the crushed Wld^S ^axon lack degeneration signs (inset 3 and 4). YFP fluorescence has been pseudo-coloured yellow with the applied confocal imaging software (Biorad LaserSharp 2000). Scale bar: 500 μm Click here for file
