# Supplementary figures and images for: The progressive nature of Wallerian degeneration in wild-type and slow Wallerian degeneration (WldS) nerves
Source: BMC Neurosci. 2005 Feb 1;6:6. doi: 10.1186/1471-2202-6-6 (PMC549193; doi:10.1186/1471-2202-6-6)

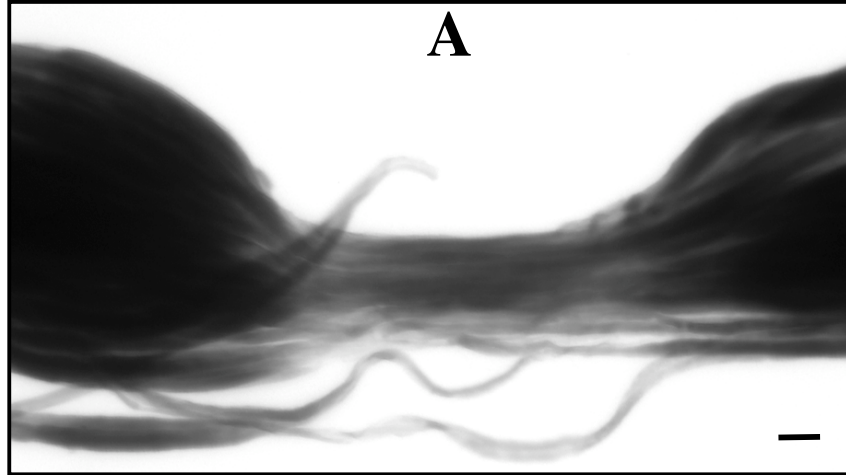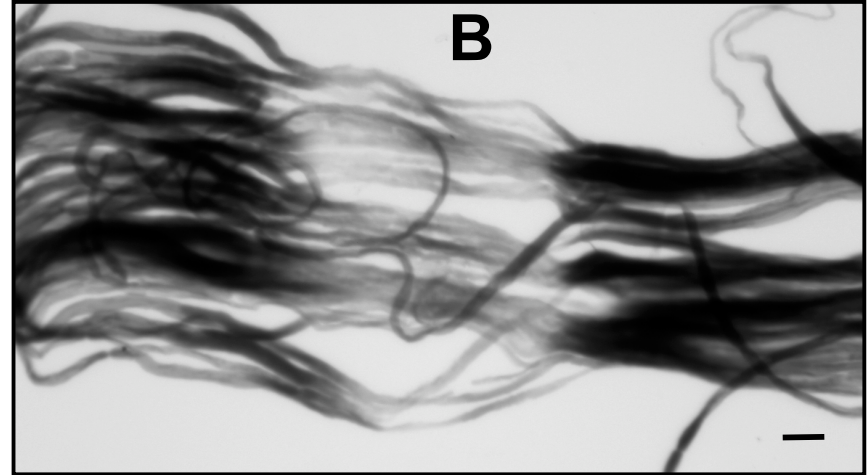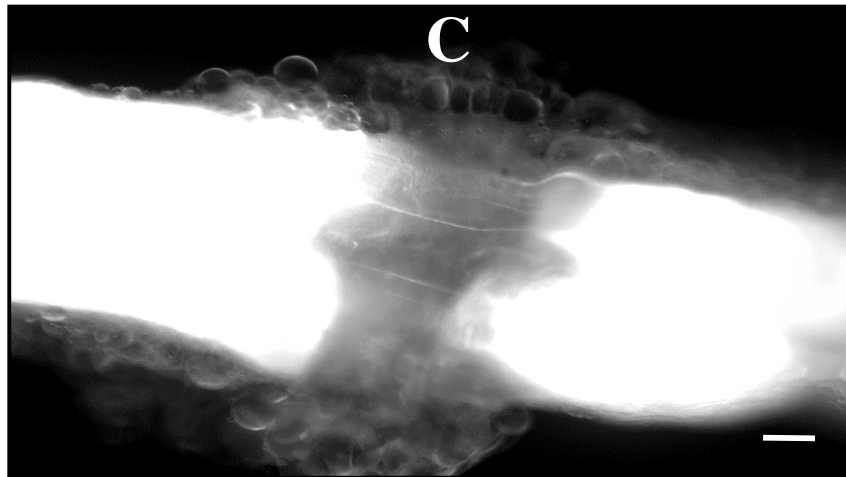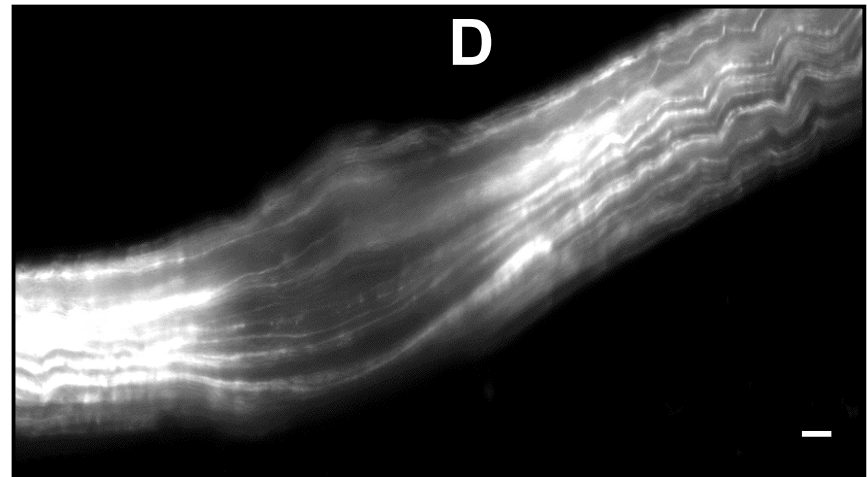

Supplement: Additional File 1 — Many nerve fibres are unbroken directly after a 30 second crush as shown in YFP-H wholemount nerves and osmificated teased fibre preparations.A-C: Sciatic nerves crushed for 30 seconds at maximum pressure and then immediately fixed and imaged by osmium staining (A, B) or YFP fluorescence (C). D: YFP-H nerve crushed for 5 seconds at maximum pressure. Nerves in (A) and (B) were partially teased apart after staining, leading to accidental breakage of a few fibres. However, the majority of fibres clearly cross the crush site unbroken. YFP signal (identifying the axoplasm) at the crush site in (C-D) is weak, probably due to squeezing of the axoplasm longitudinally out of the crush site, or quenching of fluorescence by the crushed tissue, or both. To compensate, the photographs are deliberately overexposed. There is no sign of YFP positive axoplasm escaping laterally into the extracellular space, as would be expected if the axolemma were broken. Instead, some YFP positive axons clearly cross the crush site unbroken. In (C) the overexposure would prevent any broken axons from being identified, but in (D) the signal from most or all axons fades gradually as the axon enters the crush site, rather than stopping abruptly as one would expect if the axon were broken. Scale bars: 20 μm (A, B) and 50 μm (C, D). [file 1471-2202-6-6-S1.pdf]

proximal

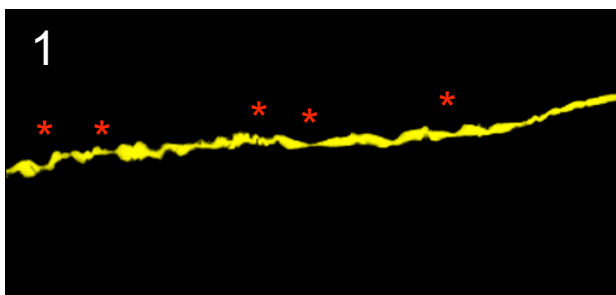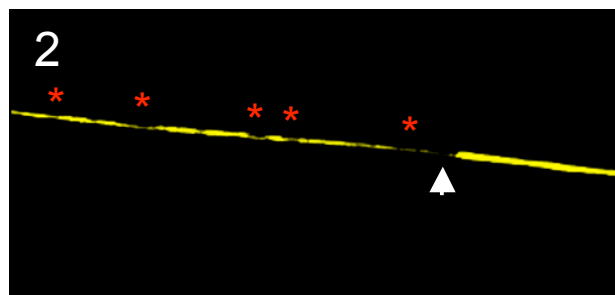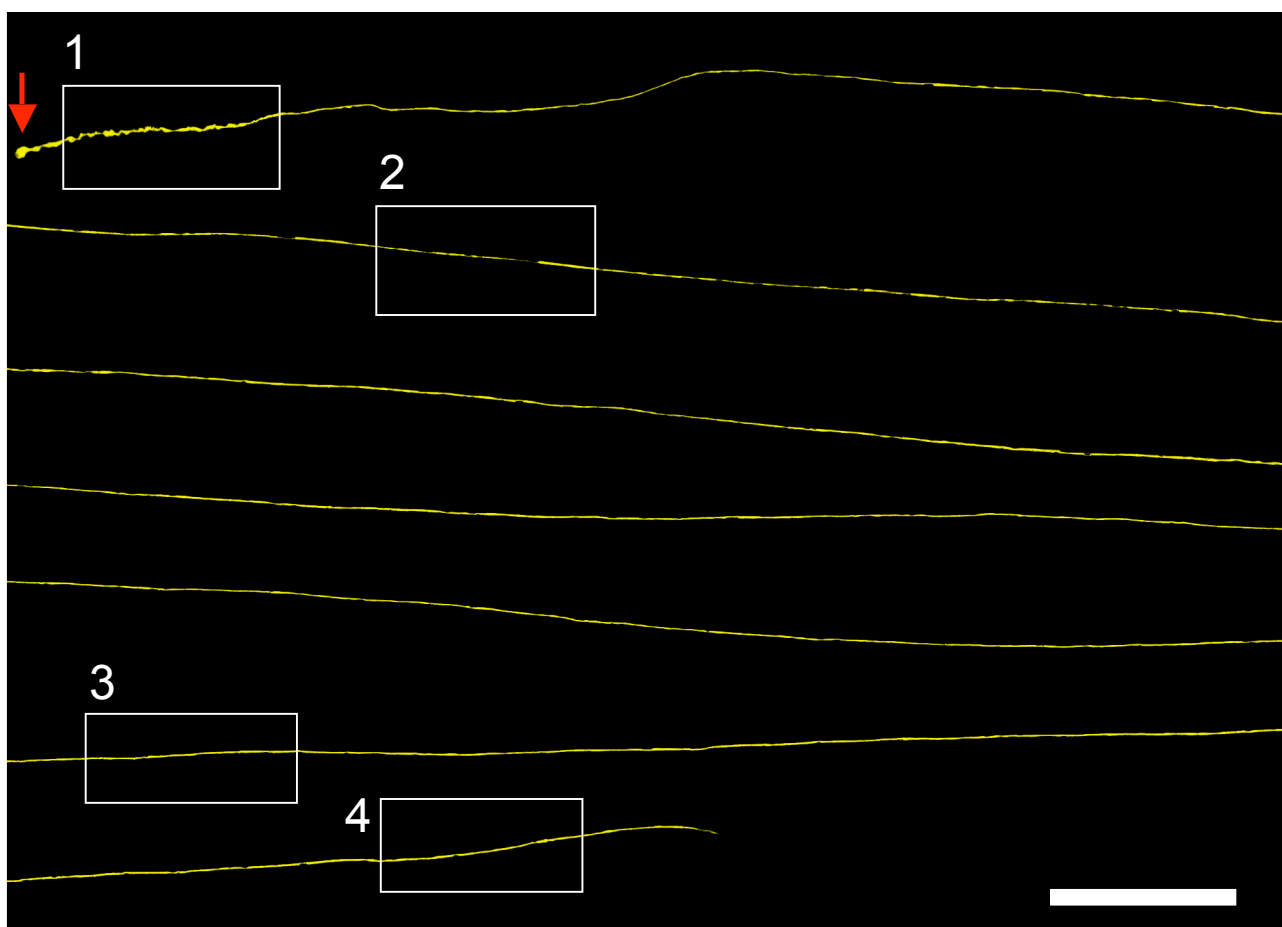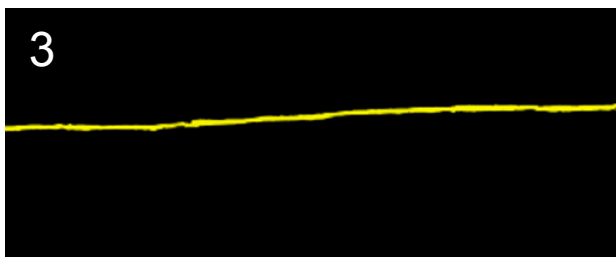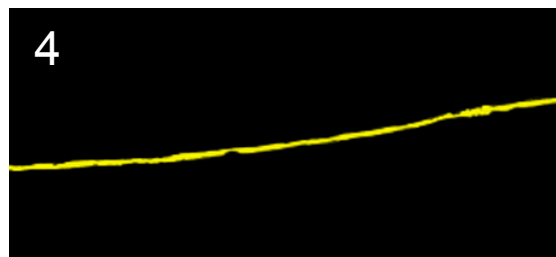

distal

Supplement: Additional File 2 — Degeneration of individual crushed WldS axons proceeds anterogradely beginning with the formation of end bulbs and axonal swellings at the most proximal end and accompanying proximal axonal atrophy. Confocal composite picture showing seven consecutive lengths (from top to bottom in overview) of the proximo-distal course of an individual YFP labelled WldS axon within a peripheral triple heterozygote nerve stump 15 days after crush injury displaying an anterograde gradient of axon degeneration. This axon exhibits an end bulb at the most proximal end (red arrow in overview) and subsequent multiple axonal swellings delimited by constrictions (red asterisks) (inset 1). Further distally these swellings disappear with remaining constrictions (red asterisks) and sporadic breaks (white arrow) (inset 2). Distal parts of the crushed WldS axon are free of degeneration signs (inset 3 and 4). Scale bar: YFP fluorescence has been pseudo-coloured yellow with the applied confocal imaging software (Biorad LaserSharp 2000). 500 μm [file 1471-2202-6-6-S2.pdf]

proximal

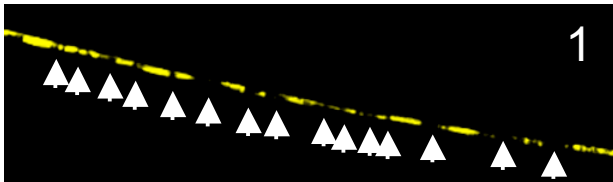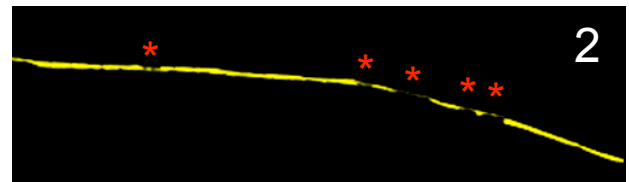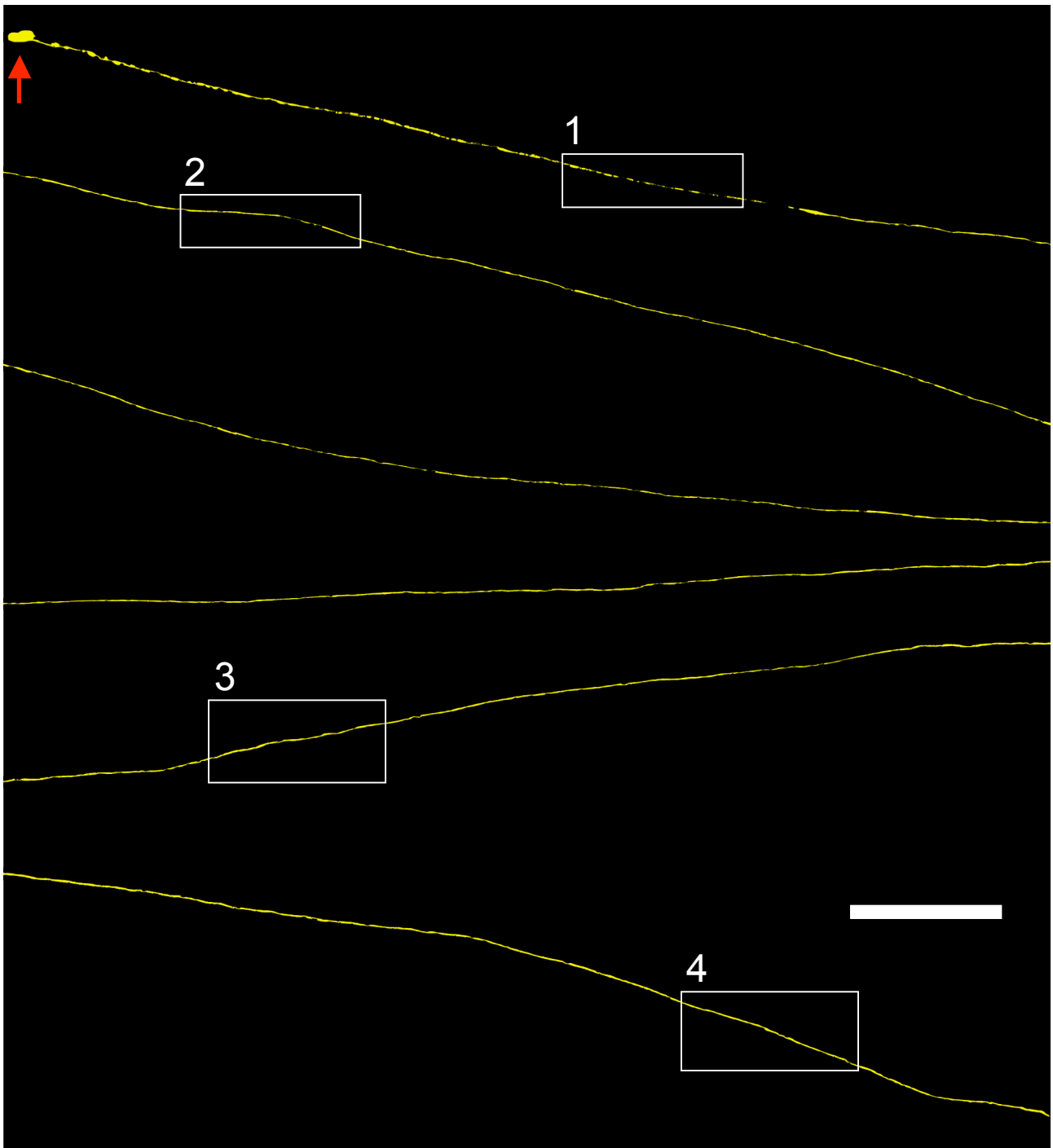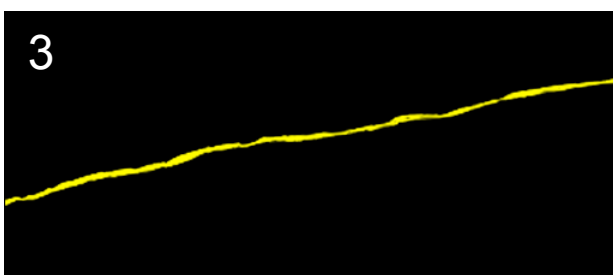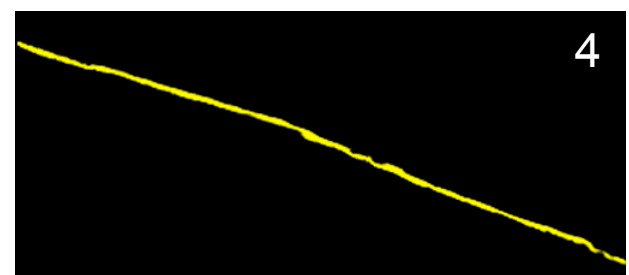

distal

Supplement: Additional File 3 — Anterograde degeneration with formation of massive end bulbs of compressed WldS axons finally includes complete proximal fragmentation. Confocal composite picture showing six consecutive lengths (from top to bottom in overview) of the proximo-distal course of an individual YFP labelled WldS axon within a peripheral triple heterozygote nerve stump 20 days after crush injury demonstrating a clearer anterograde progression of axon degeneration than in Add Fig 2.pdf. Likewise after 15 days following crush injury this picture shows a massive end bulb at the most proximal end of the distal stump (red arrow in overview) and subsequent multiple axonal swellings and constrictions. Complete axonal fragmentation is evident in inset 1 (white arrows mark axonal breaks) and gradually gives way to incomplete breakup with axonal constrictions (red asterisks) (inset 2). Distal parts of the crushed WldS axon lack degeneration signs (inset 3 and 4). YFP fluorescence has been pseudo-coloured yellow with the applied confocal imaging software (Biorad LaserSharp 2000). Scale bar: 500 μm [file 1471-2202-6-6-S3.pdf]
